# Design and artificial synthesis of gRNA library for genetic breeding of *Saccharomyces cerevisiae*

**DOI:** 10.1016/j.bidere.2026.100123

**Published:** 2026-09-28

**Authors:** Xinyi Wang, Peiyan Ma, Majda Hanioui, Zhangnian Wang, Qianqian Yu, Abdul Waheed, Kun Liu, Yan Liu, Zhenglian Xue, Ming Zhao

**Affiliations:** a Anhui Engineering Laboratory for Industrial Microbiology Molecular Breeding, College of Biology and Food Engineering, Anhui Polytechnic University, Wuhu, 241000, China; b Wuhu Green Food Industry Research Institute Co., Ltd, Wuhu, 241000, China

**Keywords:** Artificial gRNA library, In vitro transcription, CRISPR-Mediated genome mutation, *Saccharomyces cerevisiae*

## Abstract

CRISPR mediated genome engineering strategies were developed and applied for genome-wide mutagenesis. However, high-throughput gRNA libraries require design and synthesis, which limits their economy and versatility. In this study, we designed in vitro transcription strategy for artificially synthesizing random gRNA libraries, which were transformed into cells to guide the diverse mutations generated through Cas protein induced double stranded DNA breaks. As a proof of concept, the *Saccharomyces cerevisiae* strain C0 with overexpression of tHMG1 and β-carotene biosynthesis key genes was used as the original strain, and the β-carotene synthesis ability of strain C0 was increased to that of 5.8 times after three rounds of iterative mutation. Furthermore, for the mutated *S*. *cerevisiae* strain C7-143 with high-level β-carotene production, a mutant strain AgC8-174 with a 26% increase in β-carotene yield was obtained through one round of mutation. This strategy completely avoided the limitations of plasmid library, significantly shortened the experimental cycle, and improved the versatility of gRNA library across different species. The results fully demonstrated the feasibility of the technology and lays a solid foundation for its application in the field of microbial genetics and breeding.

## 1 Introduction

Under natural conditions, the mutation rate of bases in the genome of organisms is extremely low, making it difficult for evolution and breeding of organisms [1]. Therefore, mutagenesis breeding, as an artificial genome mutation technology, has been applied to the mutation evolution of organisms since the beginning of the last century, and gradually formed a complete method and theoretical system [2]. For example, physical mutagenesis techniques based on the ultraviolet mutagenesis, electromagnetic radiation, and space breeding have emerged [3]; chemical mutagenesis techniques based on the alkylating agents, base analogues, frameshift mutagens [4]; biological mutagenesis techniques based on the genome shuffling and transposon mutations [5]. These technologies have provided extremely important impetus for the establishment and development of the breeding industry, nevertheless, the drawbacks of traditional induced mutagenesis methods are increasingly problematic, such as the single mutation type, the carcinogenic mutagens, and the need for special equipment. Thus, the development of new genomic mutagenesis techniques is extremely desirable.

In recent years, the *Saccharomyces cerevisiae* 2.0 Project has advanced rapidly, aiming to create synthetic microbial strains with high applicability and versatility [[6], [7], [8]]. Accordingly, microbial genome engineering technologies have developed swiftly, laying a solid foundation for the rapid evolution of microorganisms at the whole-genome level. These technologies mainly include: (1) Synthetic Chromosome Rearrangement and Modification by LoxP-mediated Evolution (SCRaMbLE), a chromosome-engineering-based strategy that constructs yeast strains harboring synthetic chromosomes and induces chromosomal rearrangements and modifications via numerous LoxP (locus of X-over P1) sites preintroduced into the yeast genome [9,10]; (2) Multiplex Automated Genome Engineering (MAGE), a precise genome mutagenesis technique for *Escherichia coli* that employs rationally designed single-stranded oligonucleotide DNAs to introduce mutations into the *E. coli* genome through non-templated annealing with the lagging strand at the replication fork [11]; (3) Orthogonal DNA Replication system (OrthoRep), which introduces random mutations into target genes on linear plasmids using an error-prone orthogonal DNA polymerase, enabling targeted mutagenesis in both eukaryotic and prokaryotic microbes [[12], [13], [14]]; (4) Retron-mediated Genome Editing (REGES), which achieves continuous in vivo protein evolution in *E. coli* by integrating retrons, polymerase-mediated base editing, and error-prone transcription [15,16]; (5) CRISPR-mediated Genome Engineering (CMGE), including CREATE [17], CHAnGE [18], MAGSTIC [19], and other approaches. These methods construct gRNA plasmid libraries of defined size for intracellular gRNA expression, guide double-stranded DNA cleavage by Cas proteins, and generate extensive site-specific mutations in microbial genomes via homologous recombination repair with synthetic donor DNA templates. Although the aforementioned mutagenesis tools enable genome editing in diverse microorganisms, the development of robust random and diverse mutagenesis technologies applicable across a broad range of microbial species remains challenging [20,21].

DNA double-strand breaks (DSBs) represent severe DNA lesions and are the major drivers of diverse genomic mutations in organisms. To survive, cells promptly activate robust DNA repair pathways to mend the broken DNA, which concurrently generates mutations including base substitutions, insertions and deletions of bases or DNA fragments, as well as translocations and rearrangements [22,23]. In our previous work [24], we employed primers containing 20 degenerate bases to amplify random gRNA expression cassettes in vitro, which were recombined with plasmid backbones in vivo in *S. cerevisiae* to generate gRNA plasmid libraries and produce gRNA pools. These gRNAs guided Cas protein to induce multiple double-strand DNA breaks (DSBs) in the *S. cerevisiae* genome, and triggered diverse genomic mutations including base insertions, deletions, duplications, inversions, and chromosomal rearrangements during the non-homologous end joining repair. However, this strategy suffered from common limitations of traditional gRNA-mediated genome engineering (GMGE) technologies, such as limited gRNAs due to plasmid copy number and poor library versatility due to incompatible expression elements. To address these issues, here, we designed in vitro transcription strategy for artificially synthesizing random gRNA libraries. Using a β-carotene producing *S. cerevisiae* strain as a model, this strategy enabled rapid and highly efficient strain evolution within an extremely short time. This strategy will provide a new route for the broad application of CMGE in microbial genetic breeding.

## 2 Materials and methods

### 2.1 Strains and plasmids

The *S. cerevisiae* strains used in this study included the original strain C0 and the high-yield β-carotene mutant strain C7-143 (Supplementary Table S1). Strain C0 was derived from *S. cerevisiae* BY4741. Its genome was integrated with expression cassettes of key genes in the mevalonate (MVA) pathway (*tHMG1*) and β-carotene synthesis-related genes (*crtE*, *crtYB*, and *crtI*), and it harbored the plasmid pCas9-NG (Supplementary Table S2) expressing the Cas9-NG mutant protein, which recognizes NG protospacer adjacent motif (PAM) sequences.

### 2.2 Primers、reagents and growth conditions

Primers used in this study were listed in Supplementary Table S3. A long primer library containing 20 degenerate bases was designed and synthesized, with the sequence arranged from the 5′ end to the 3′ end as follows: T7 promoter sequence, gRNA guiding sequence with 20 degenerate bases, and gRNA scaffold sequence. The degenerate region (N20) was synthesized using equimolar-mixed phosphoramidites by GenScript Biotech Corporation (Nanjing, China). Two specific primers for PCR amplification were also prepared. T7 RNA polymerase, PCR kits, RNA purification kits, NanoDrop spectrophotometers, reagents for agarose gel electrophoresis, and reagents for β-carotene extraction and detection were all purchased from conventional biological reagent suppliers. The components of yeast extract peptone dextrose (YPD) medium were as follows: 10 g/L yeast extract, 20 g/L peptone, and 20 g/L glucose. 20 g/L agar was added to prepare solid medium. *S. cerevisiae* strains were cultured at 30 °C with shaking at 220 rpm in YPD medium. All experimental reagents were of analytical or biological grade.

### 2.3 Construction of long primer library with 20 degenerate bases and gDNA_20N_ template library

Based on the structural characteristics of gRNA, a long primer library was designed. Its sequence consisted of a T7 promoter, a 20-nucleotide degenerate gRNA targeting region (N represents random distribution of A/T/C/G bases), and a gRNA scaffold from the 5′ to 3′ terminus. Using the long primer library as the template, PCR amplification was performed with specific upstream and downstream primers to construct the gDNA_20N_ template library. The 50 μL PCR reaction system contained 1 μL of the template primer library, 1.5 μL each of the upstream and downstream primers, 25 μL of PCR Mix, and 21 μL of sterile deionized water. The PCR reaction conditions were set as follows: initial denaturation at 98 °C for 5 min; 30 cycles of denaturation at 98 °C for 10 s, annealing at 40.6 °C for 5 s, and extension at 68 °C for 10 s; final extension at 68 °C for 5 min, followed by preservation at 4 °C. After verification by agarose gel electrophoresis, the PCR products were purified and recovered to obtain the purified gDNA_20N_ template library.

### 2.4 In vitro transcription and purification of random gRNA_20N_ library

The purified gDNA_20N_ template library was used as the template for in vitro transcription catalyzed by T7 RNA polymerase to generate the random gRNA_20N_ library. The transcription reaction system was configured in accordance with the manufacturer's instructions for the T7 RNA polymerase kit, and the mixture was incubated at 37 °C for 4 h to ensure sufficient transcription. After the reaction, residual template DNA and impurities were removed using an RNA purification kit to obtain purified gRNA_20N_. The uniformity of the gRNA library was detected by agarose gel electrophoresis, and the purity (OD_260_/OD_280_ ratio) and concentration were determined using a NanoDrop spectrophotometer. Qualified libraries were required to meet the following criteria: OD_260_/OD_280_ = 2.0–2.2 and concentration ≥ 1000 ng/μL. Meanwhile, cloning and sequencing were conducted to analyze the random distribution of the 20 degenerate bases in the gDNA_20N_ library and verify its randomness.

### 2.5 Preparation of yeast competent cells and electrotransformation

*S. cerevisiae* strains C0 and C7-143 were cultured to the logarithmic growth phase (OD_600_ = 0.6–0.8). Cells were collected by centrifugation, washed twice with pre-chilled sterile water and twice with pre-chilled 1 mol/L sorbitol solution, and finally resuspended in pre-chilled 1 mol/L sorbitol solution to prepare competent cells, which were placed on ice for subsequent use. A total of 100 μL competent cells were mixed with purified gRNA_20N_ library, incubated on ice for 30 min, and then transferred into a pre-chilled electroporation cuvette. Electrotransformation was performed under the parameters of 2.5 kV voltage, 25 μF capacitance and 400 Ω resistance. Immediately after electroporation, 0.5 mL YPD liquid medium was added, and cells were cultured at 30 °C with shaking at 200 rpm for 2 h for recovery. Strains C0 and C7-143 were electroporated under identical conditions.

### 2.6 Optimization of electrotransformation conditions and detection of strain mortality

To determine the optimal electrotransformation dosage of the gRNA_20N_ library, a dosage gradient (0.5 μg, 1.0 μg, and 1.5 μg) was set for electrotransformation into C0 competent cells. After recovery, the bacterial solution was serially diluted and spread onto YPD solid medium, followed by incubation at 30 °C for 48 h. The number of colonies was counted to calculate the strain mortality, which was calculated as follows: Mortality = [(Colony number of blank control group-Colony number of gRNA-treated group)/Colony number of blank control group]×100%. The gRNA dosage corresponding to the highest mortality was selected as the optimal condition for subsequent iterative mutagenesis.

### 2.7 AgLAGM-mediated iterative mutagenesis and mutant screening of *S. cerevisiae*

The optimized dosage of gRNA_20N_ library (1.5 μg) was transformed into C0 competent cells. After recovery, the bacterial liquid was spread on YPD solid medium and cultured at 30 °C for 48 h, with the colony number controlled at 200–300 per plate for convenient single-colony picking. Preliminary screening was carried out according to the orange-yellow color depth of colonies, as the β-carotene yield was positively correlated with colony color intensity. Single colonies with significantly deeper color than the original strain were selected for shake-flask fermentation. The β-carotene yield was quantified by HPLC. Mutants with a yield increase of more than 20% compared with the original strain were defined as effective positive mutants, and the positive mutation rate was calculated. The superior mutant from each round was selected as the starting strain for the next round of mutagenesis, and a total of three rounds of iterative mutagenesis were performed to detect the β-carotene yield of the top mutant in each round. For the high-yield strain C7-143, the gRNA_20N_ library was introduced into its competent cells under the same optimized electrotransformation conditions. After incubation at 30 °C for 48 h, more than 10^4^ colonies were preliminarily screened by visual observation of colony color. A total of 216 strains were selected for test-tube fermentation preliminary screening. Strains with higher β-carotene yield than C7-143 were further verified by shake-flask fermentation, and their yields were determined to screen the ultimate high-yield mutant.

### 2.8 Analysis of β-Carotene yield

The screened mutants and original strains were inoculated into 50 mL YPD liquid medium and cultured at 30 °C with shaking at 220 rpm for 72 h. Cells were collected by centrifugation, washed twice with sterile water, and freeze-dried to constant weight to calculate the cell dry weight (CDW). Collect 1 mL of bacterial culture into a 1.5 mL centrifuge tube, centrifuge at 8000 r/min for 2 min at 4 °C, and discard the supernatant. Wash the cell pellet with 1 mL of sterile water, centrifuge at 8000 r/min for 2 min, and remove the supernatant. Add 1 mL of 3 mol/L HCl to fully resuspend the cells, perform a boiling-water bath for 3 min, then immediately place the tube on ice for 2 min. Centrifuge and discard the HCl supernatant. Wash the pellet once with 1 mL of sterile water and centrifuge to remove the supernatant. Add 1 mL of acetone and vortex on a vortex mixer for 5 min until the cells become colourless [25]. High-performance liquid chromatography (HPLC) was applied to identify and quantify carotenoids synthesized by engineered yeasts. The extraction solution was filtered through a membrane and transferred into liquid-phase vials for HPLC detection. An Agilent ZORBAX SB-AQ chromatographic column (5 μm, 4.6 mm × 250 mm) equipped with a UV/Vis detector was used. The mobile phases consisted of eluent A (90% acetonitrile aqueous solution) and eluent B (methanol-isopropanol, 3:2, v/v), with a flow rate of 1 mL/min. The detection wavelength was set at 450 nm and the column temperature was maintained at 30 °C. The gradient elution procedure was as follows: 0–15 min, 100%–10% eluent A and 0%–90% eluent B; 15–30 min, 10% eluent A and 90% eluent B; 30–35 min, 10%-100% eluent A and 90%-0% eluent B [24].

## 3 Results and discussion

### 3.1 Design of gRNA_20N_ in vitro for microbial genetic breeding

Microbial double-strand break (DSB) repair comprises multiple repair systems, including homologous recombination (HR), non-homologous end joining (NHEJ), and microhomology-mediated end joining (MMEJ). Except for HR repair, NHEJ and MMEJ need not homologous fragment and can trigger non-fidelity repair at DSB sites, which produce diverse mutation types [26,27]. Therefore, DSB repair is suitable for high-frequency diversified mutagenesis in microbial genomes. Microbial genome can just trigger DSB under major damage, which is difficult to achieve through traditional physicochemical mutagenesis techniques, and thus the high frequency techniques for inducing DSB in microbial genomes are crucial for microbial breeding [28,29].

Over the past decade, the CRISPR system, with its efficient and precise DNA-breaking capability, has been developed into a gene-editing tool and applied to various microorganisms such as bacteria, actinomycetes, and yeast [30]. In the CRISPR system, the Cas protein, a kind of nuclease cleaves, binds to the tracrRNA region of the guide RNA (gRNA) and targets to the designed genomic sites to trigger DBS under the crRNA region of gRNA [31]. Therefore, CRISPR system can be used to induce high frequency DSB with a abundant gRNA library. Herein, a strategy for random mutagenesis across the whole microbial genome through CRISPR system was proposed. The core concept was as follows: a gRNA library containing 20 degenerate bases (corresponding to the gRNA guiding region) was used to guide Cas proteins to randomly break the microbial genome, and the diversified genomic mutations were produced during the non-faithful DNA repair. As a proof of concept, in our previous work [24], long primers containing 20 degenerate bases were used for PCR amplification and a random gRNA expression cassette library with a theoretical complexity of 4^20^ (about 10^12^) was achieved, which could target all of the microbial genome. Using a β-carotene-producing *S. cerevisiae* strain as a model, we demonstrated that the random gRNA expression cassette library could be cycled to random gRNA plasmid library and transcribed into gRNA library through co-electrotransformation with plasmid skeleton. Except that, random gRNA library could guide Cas protein to randomly cleave the model genome, inducing various mutations including base insertions, deletions, duplications, inversions, and chromosomal rearrangements during the non-fidelity repair [32]. Such a wide spectrum of mutation types not only overcomes the limitation of single-type mutations in the traditional genome mutagenesis techniques but also offers operational safety and simplicity, without requiring specialised mutagenesis equipment.

Despite these advantages, this mutagenesis system still has several drawbacks: (1) Construction of a large-scale gRNA plasmid library is required, which involves complex procedures and difficult quality control. For example, achieving a complete library would demand a plasmid clone scale of 4^20^, which is impractical. (2) Plasmid copy number restricts the diversity of gRNAs synthesized within the cell, thereby limiting the number of genomic sites that can be mutated. (3) Plasmid skeletons and gRNA expression cassette elements are different between different hosts, thus meaning that a gRNA library constructed for one host is not universally compatible in others. To address these issues, here, artificial synthesis of gRNA library was achieved through in vitro transcription strategy. Firstly, the random gRNA library expression cassette located downstream of the T7 promoter was obtained through PCR amplification with a long-primer pool containing 20 degenerate bases. Secondly, amplified gRNA library cassette was transcribed into random gRNA library under T7 RNA polymerase in vitro. Then, the random gRNA library was introduced by electrotransformation into a microbial strain expressing Cas protein, establishing a novel genome mutagenesis system (Fig. 1), herein named AgLAGM (Artificial gRNA Library Assisted Genome Mutation). Finally, Cas protein can trigger genomic DSBs at various loci to generate diversified genomic mutations during the non-fidelity repair and mutants was achieved through screening. Compared with other CMGE methods, AgLAGM directly synthesises the artificial random gRNA library (artificial gRNA_20N_) in vitro, circumventing the numerous constraints associated with constructing large-scale plasmid libraries. Furthermore, this gRNA library is universally applicable for different hosts.

**Fig. 1:**
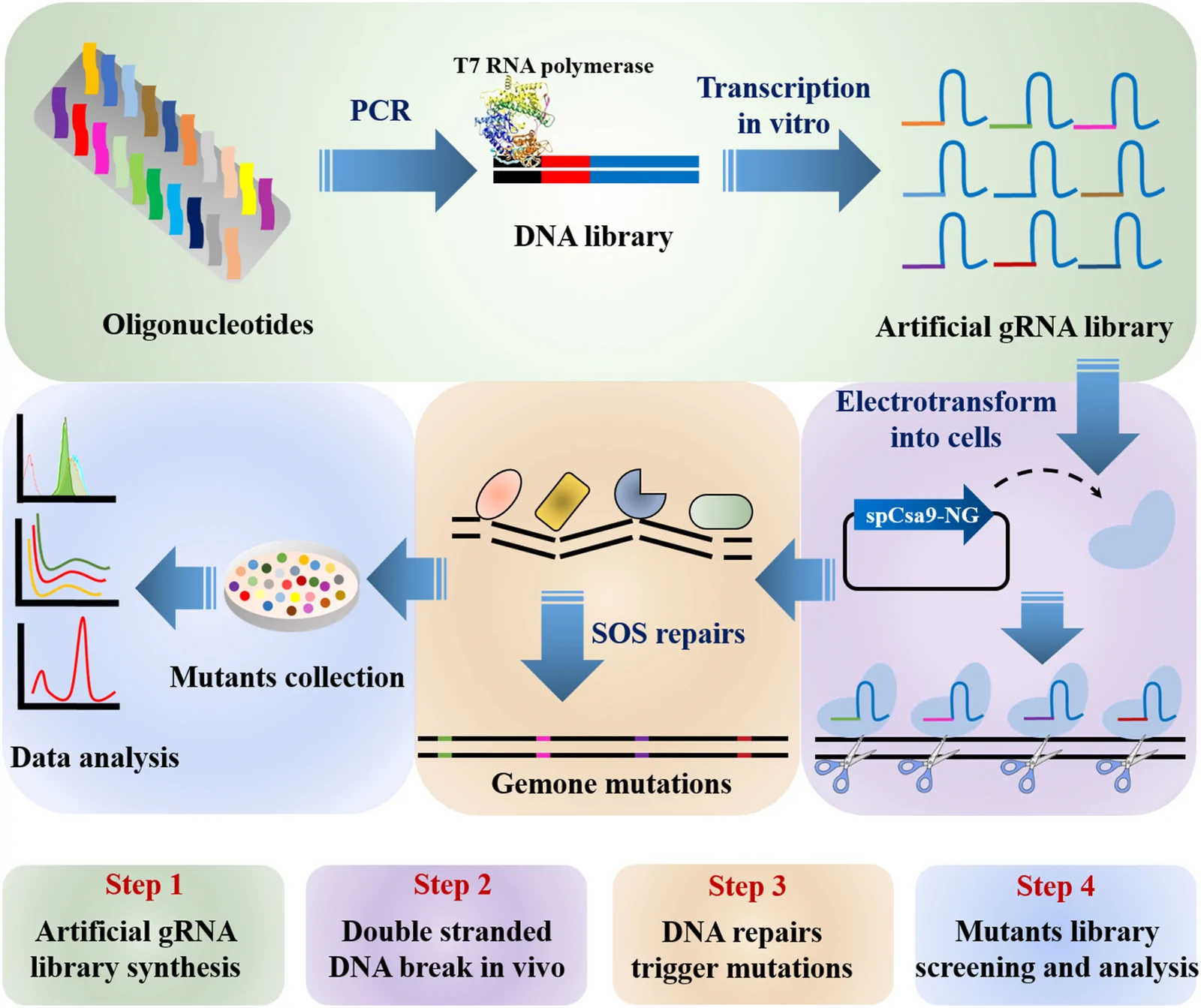
Random gRNA libraries by in vitro transcription for targeted genome evolution of β-carotene-producing yeast. Step1: Artificial gRNA library synthesis. Step2: Double stranded DNA break in vivo. Step3: DNA repairs trigger mutations. Step4: Mutants library screening and analysis.

### 3.2 In vitro synthesis of artificial gRNA_20N_

Currently, the strategies for the synthesis of random gRNA library in vitro has not been reported. Therefore, creating a novel strategy for the synthesis of high-abundance artificial gRNA_20N_ is crucial for the establishment of the AgLAGM system. Considering that the SELEX (Systematic Evolution of Ligands by Exponential Enrichment) technique is widely used for the construction of riboswitch libraries, the lengths of which can reach 60–150 bases with random regions of 20–50 bases, therefore, SELEX would be fully suitable for the in vitro synthesis of artificial gRNA_20N_ [33]. Herein, We designed and synthesized a long primer library (Fig. 2A), which contained T7 RNA polymerase binding region, degeneracy bases region, and gRNA scaffold region. After the PCR amplification of the synthesized primer library with forward and reverse primers, the template, gDNA_20N_ library, for in vitro transcription of artificial gRNA_20N_ was obtained. To ensure the abundance of transcribed gRNA_20N_, the randomness of 20 degenerate bases in gDNA_20N_ should be verified before the in vitro transcription. Through the construction of about 30 clones and sequencing analysis, the occurrence probability of the 20 degenerate bases was demonstrated to be nearly uniform (Fig. 2B–Supplementary Table S4), resulting in synthesizing high-abundance artificial gRNA_20N_. As shown in Fig. 2B, the non-uniform randomness observed at certain sites results from the limited sample number, whereas a sufficient sample size would yield fully uniform randomness at all sites.

**Fig. 2:**
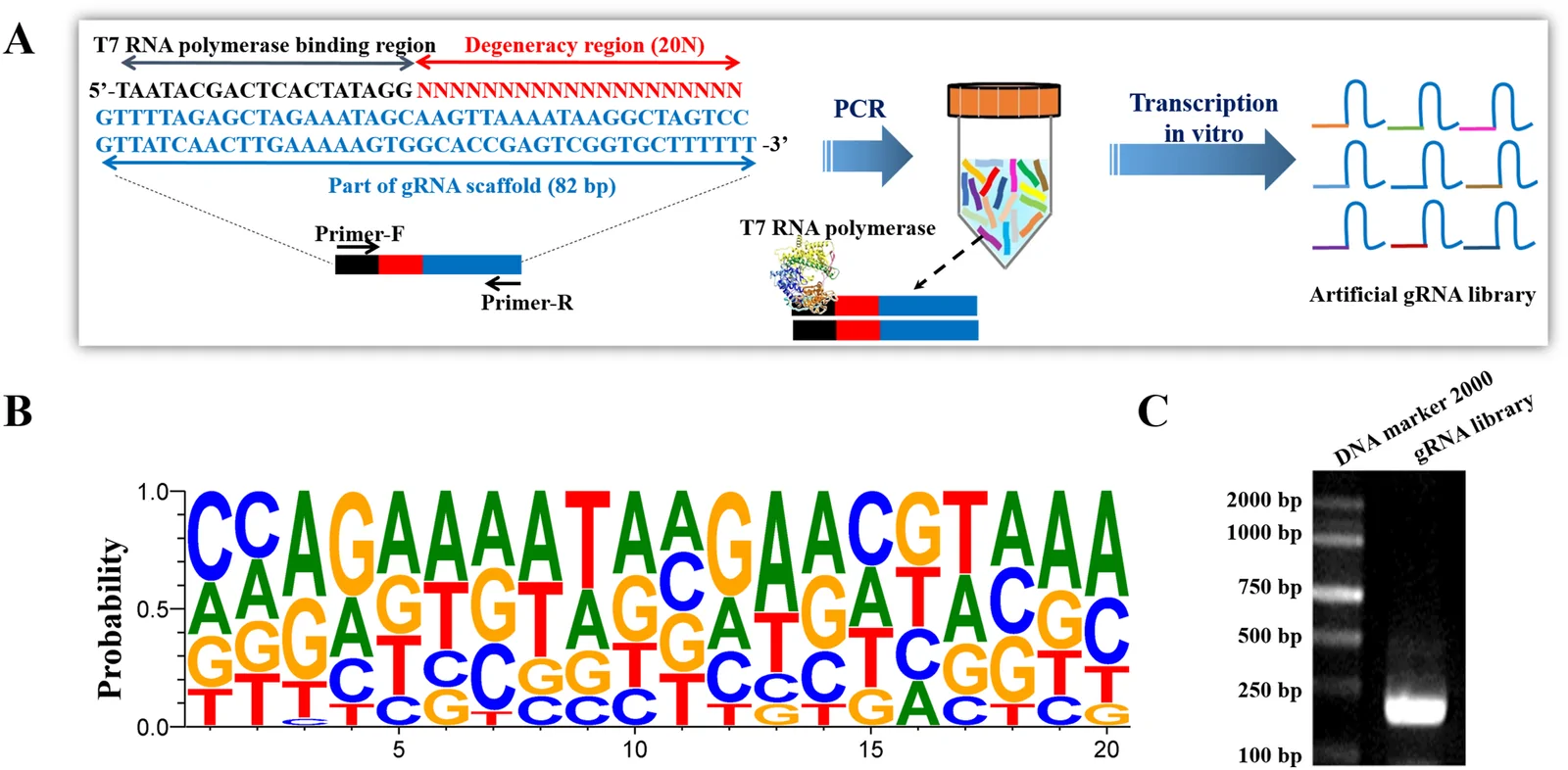
Design and synthesis of artificial gRNA_20N_. A. Schematic for synthesis of gRNA_20N_ in vivo. B. Randomization analysis of the 20 bp consecutive degeneracy bases of gRNA_20N_. The consensus sequence was analyzed using WebLogo 3: Create (https://weblogo.threeplusone.com/create.cgi). C. Quality analysis of synthesized gRNA_20N_ by agarose gel electrophoresis.

T7 RNA polymerase can initiate the synthesis of RNA complementary to one strand of template DNA downstream of the T7 promoter, enabling rapid production of large quantities of RNA molecules. The transcribed RNA can be widely applied in multiple research fields, including studies on RNA structure and function, RNase protection assays, probe hybridization, RNA interference, microinjection, and in vitro translation [[34], [35], [36]]. Herein, using the synthesized random gDNA_20N_ library as template, the in vitro transcription strategy with T7 RNA polymerase was employed to synthesize artificial gRNA_20N_. Electrophoresis results revealed a single band, demonstrating high homogeneity of the synthesize artificial gRNA_20N_ (Fig. 2C). Analysis of the purified random gRNA_20N_ library using a NanoDrop spectrophotometer gave an OD_260_/OD_280_ ratio of 2.11 and a concentration of 1739 ng/μL, indicating high purity and concentration of the purified artificial gRNA_20N_. These results confirm that the synthesis of high-quality gRNA library via the T7 RNA polymerase-based in vitro transcription strategy is fully feasible, which laid the foundation for guide Cas protein to induce mutations of the microbial genome in vivo.

### 3.3 Validation of the evolutionary capacity of AgLAGM in *S. cerevisiae*

In our previous research, we had demonstrated that random gRNA expressed plasmid library can transcribed gRNA library can guide Cas protein to trigger genomic diversified mutations comprised indels, duplications, inversions, and chromosomal rearrangements in *S. cerevisiae*. Herein, a microbial model, *S. cerevisiae* C0 with the capacity of to produce β-carotene, was constructed. This strain was derived from *S. cerevisiae* BY4741 by integrating the expression cassettes of *tHMG1*, a key gene in the MVA pathway, and the β-carotene biosynthesis indispensable genes *crtE*, *crtYB*, and *crtI* into the genome [24]. In addition, C0 strain contained the recombinant plasmid pCas9-NG, which can express the Cas9 mutant recognizing the NG PAM site.

To verify that the AgLAGM system is applicable to microbial genetic breeding, the previously constructed *S. cerevisiae* strain C0 was still used as model strain. Artificial gRNA_20N_ of AgLAGM system need be transformed into cells, therefore, high efficient RNA transformation technology is crucial for improving the efficiency of microbial genome evolution in the AgLAGM system. Electroporation technology has been reported to deliver RNA into microbial cells [[37], [38], [39]], herein, we optimized the parameters of electroporation to regulate the genomic mutation efficiency of strain C0 mediated by the AgLAGM system. Considering that unrepaired DNA double-strand breaks can lead to cell death, the electroporation parameters were evaluated by strain lethality. The number of artificial gRNA_20N_ introduced into cells is a critical parameter that determines the assault effect of SpCas9-NG on the genomes and survival rates of cells. Except that, voltage and number of competent cells would also affect RNA entry into cells. As showing in (Fig. 3A), the results indicated that the number of surviving transformants with artificial gRNA_20N_ was significantly lower than that artificial gRNA_20N_, demonstrating artificial gRNA_20N_ can guide SpCas9-NG to trigger DSB and death. In addition, fewer competent cells are required for achieving a high lethality rate, which is likely to ensure sufficient RNA enters individual cells. Finally, the highest mortality up to 25.3% with 1.5 μg of the gRNA_20N_ electroporated into 10^6^ C0 competent cells under the voltage condition of 2.5 KV. Therefore, this optimized parameters of AgLAGM system were used for genomic evolution of C0.

**Fig. 3:**
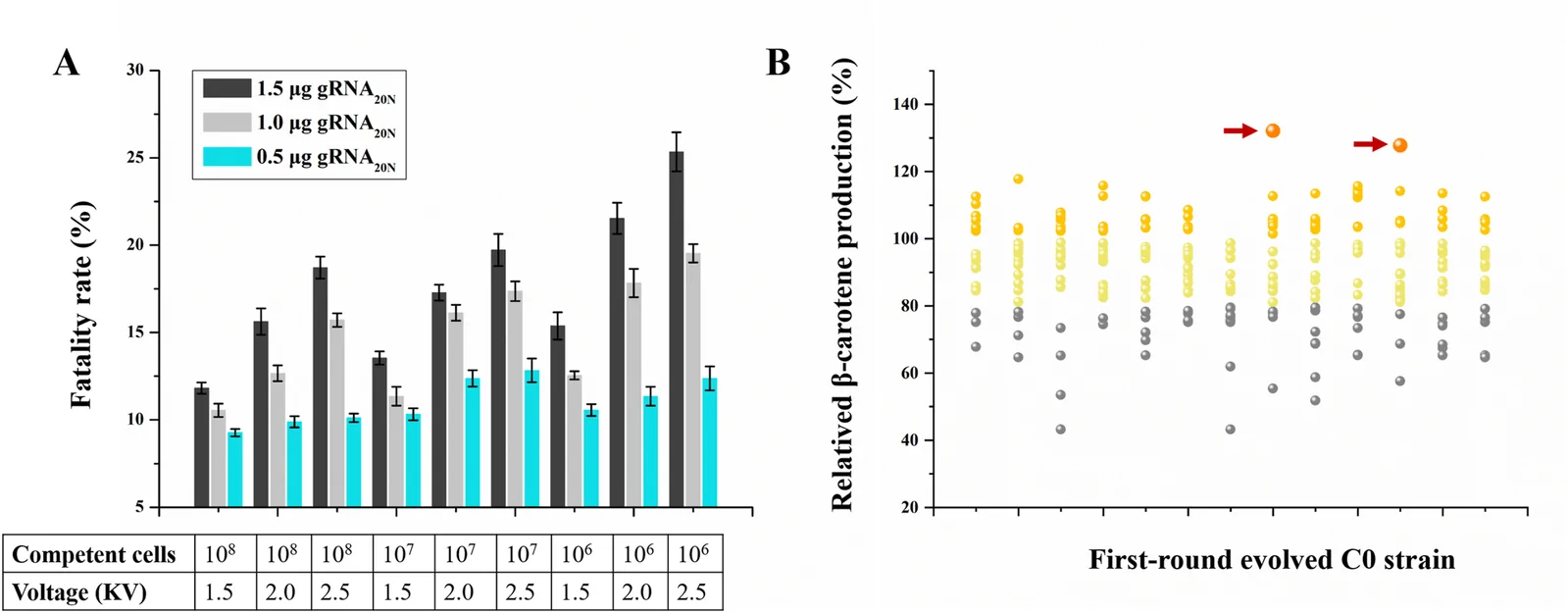
Evaluation of the AgLAGM System for genome evolution of *S*. *cerevisiae*. A. Optimization of AgLAGM condition via fatality rate analysis. Error bars show the standard deviation from three independent experiments. B. Positive mutation rate analysis of AgLAGM via β-carotene production analysis of *S. cerevisiae* C0 mutants.

Positive mutation rate serves as a key factor for evaluating microbial genetic breeding strategies. In view of the fact that differences in β-carotene production in C0 cells lead to colonies displaying orange-yellow coloration of varying intensities, in this study, the color shade was adopted as the screening criterion for high-yield β-carotene mutant strains (Supplementary Fig. S1). For C0 competent cells subjected to electroporation with the artificial gRNA_20N_, the number of colonies on each plate was controlled within the range of 200 to 300 to ensure sufficient single colonies for easy picking. To verify the positive mutation rate of the AgLAGM system on β-carotene production in strain C0, we determined the β-carotene content and calculated the positive mutation rate of all 273 mutants isolated from a plate of the first-round evolved C0 strains. Mutants with a β-carotene yield increase of more than 20% relative to the original strain were defined as effective positive mutants. The results revealed that two mutants exhibited a yield increase exceeding 20%, corresponding to a positive mutation rate of 7.3‰ (Fig. 3B), illustrating the reliability and efficiency of the AgLAGM system in directed evolution of *S*. *cerevisiae*. Among all obtained mutants, strain AgC1-151 showed the highest β-carotene production, which was 32% higher than that of the parental strain C0.

### 3.4 Validation of iterative evolution capability of AgLAGM

To verify the evolutionary capacity of the AgLAGM system, we performed iterative evolution for C0 strain. Competent cells of strain AgC1-151 were prepared and re-electroporated with the artificial gRNA_20N_ to screen mutants with further improved β-carotene biosynthesis. The number of mutants on each plate was controlled about 200 and strains with distinctly enhanced orange-red pigmentation were selected. The results showed that the optimal mutant AgC2-11 obtained from the second round exhibited a β-carotene production of 10.55 mg/g CDW, which was 3.91-fold and 2.95-fold higher than that of the original strain C0 and AgC1-151, respectively (Fig. 4A, Supplementary Fig. S2). Subsequently, a third round of iterative evolution was conducted using AgC2-11, and the final mutant AgC3-17 was acquired with a β-carotene production of 15.69 mg/g CDW, corresponding to 5.80-fold and 1.48-fold increases relative to C0 and AgC2-11 (Fig. 4A, Supplementary Fig. S3). Owing to the elimination of procedures including construction of gRNA_20N_ expression plasmid libraries and intracellular plasmid knockout, the AgLAGM system enabled three rounds of iterative evolution in *S*. *cerevisiae* within only two weeks and effectively improved strain performance, thereby confirming its great potential for microbial mutation breeding.

**Fig. 4:**
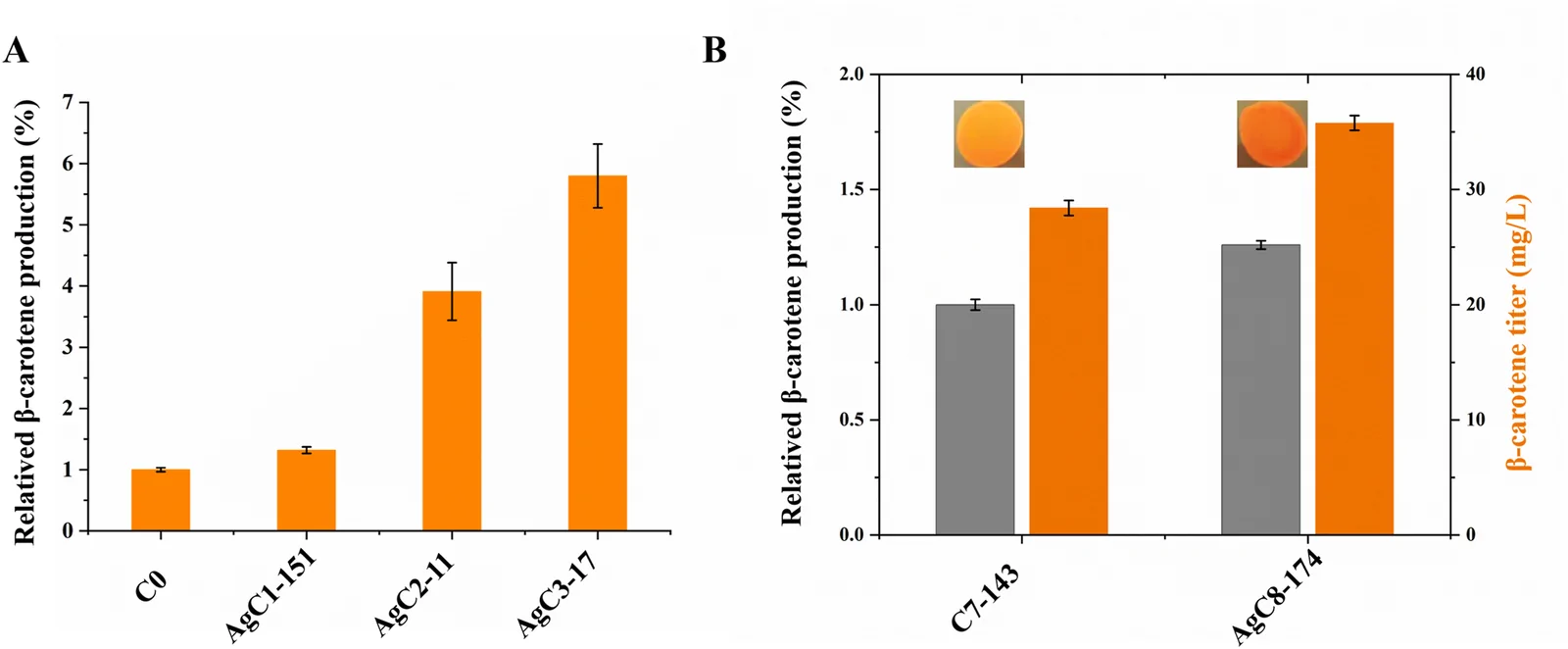
β-carotene production analysis of iteratively evolved mutants. A. β-carotene production analysis of screened C0 mutants by three rounds of iterative evolution. B. β-carotene production analysis of screened mutants of β-carotene high level production strain C7-143. All the strains were grown in YPD media at 30 °C for 72 h. The grey bars represent the relative β-carotene yield of strains, and the orange bars represent the actual β-carotene yield of strains. Error bars show the standard deviation from three independent experiments.

To further explore the evolutionary efficiency of the AgLAGM system toward high-yield strains, we used high β-carotene-producing (28.4 mg/g CDW) *S*. *cerevisiae* strain, C7-143, representing one of the highest levels of single-cell β-carotene synthesis reported,as the model. Accordingly, conventional microbial genome mutagenesis strategies, including CARM, hardly achieve further β-carotene production improvement C7-143. Here, the aforementioned iterative evolution strategy was applied to competent cells of C7-143 via electroporation of the artificial gRNA_20N_, and more than 10^4^ mutants were screened for achieving higher β-carotene production strains. Given the inherently deep colony pigmentation of C7-143 that hinders visual discrimination, preliminary screening was firstly carried out, and a total of 216 mutants were picked for shake-tube fermentation and β-carotene production analysis. After the preliminary screening, nine mutants displayed higher β-carotene production than the parental strain C7-143 (Fig. 4B, Supplementary Fig. S4). Furthermore, AgC8-174 as the superior mutant was verified with the maximum β-carotene production reaching 35.78 mg/g CDW, which was 1.26 times that of C7-143 (Fig. 4B). This titer has reached the highest documented level of β-carotene accumulation in *S*. *cerevisiae* (Table 1).

**Table 1:** β-carotene production by *Saccharomyces cerevisiae* cell factories.

stains	β-carotene titer (mg/g DCW)	initial medium	engineering strategy	refs
*S*. *cerevisiae*	17.7	SD and SG	Point-mutation and CRISPR-Cas-mediated structural mutagenesis, combined with H_2_O_2_-resistance screening	[40]
*S*. *cerevisiae*	12.71(total carotenoids)	YPD medium	integration of β-carotene pathway into synthetic chassis, followed by SCRaMbLE-mediated genome rearrangement	[9]
*S*. *cerevisiae*	20.5	YPD medium	H_2_O_2_-driven adaptive laboratory evolution leveraging β-carotene antioxidant property; beneficial mutations identified by whole-genome sequencing and validated via reverse-engineering	[41]
*S*. *cerevisiae*	28.4	YPD medium	integration of *crtE/crtYB/crtI* and *tHMG1*; plasmid-borne random gRNA library guides Cas9-NG for genome-wide DSBs (CARM)	[24]
*S*. *cerevisiae*	35.78	YPD medium	integration of *crtE/crtYB/crtI* and *tHMG1*; in vitro-transcribed gRNA_20N_ library electroporated to guide Cas9-NG for genome-wide DSBs (AgLAGM)	This study

For the high-yield strain C7-143 trapped in a performance bottleneck (representing a high baseline for β-carotene synthesis), traditional mutagenesis and genome-editing strategies barely enabled further titer improvement. By contrast, AgLAGM could still introduce novel genomic mutations to break this bottleneck, and the resulting mutant achieved the highest reported level of β-carotene accumulation in *S*. *cerevisiae*.

### 3.5 Genetic stability assay of strain AgC8-174

To verify the genetic stability of strain AgC8-174, successive generations of AgC8-174 was performed in the absence of selective pressure. Strains of each generation were streaked on agar plates, and single colonies were picked from plates of each generation for shake-flask fermentation and the β-carotene yield was subsequently determined. Growth assay on YPD agar plates exhibited that no significant difference in β-carotene yield was finally observed, indicating that the phenotype was not lost (Fig. 5A). Except that, the β-carotene yield of each strains was almost identical, fully demonstrating the stability of mutant strain AgC8-174 (Fig. 5B). Collectively, the AgLAGM system possessed prominent advantages in efficient, convenient and rapid laboratory-directed evolution, and can effectively avoid the inherent limitations of traditional mutagenesis and chromosomal multi-site genome editing (CMGE) technologies.

**Fig. 5:**
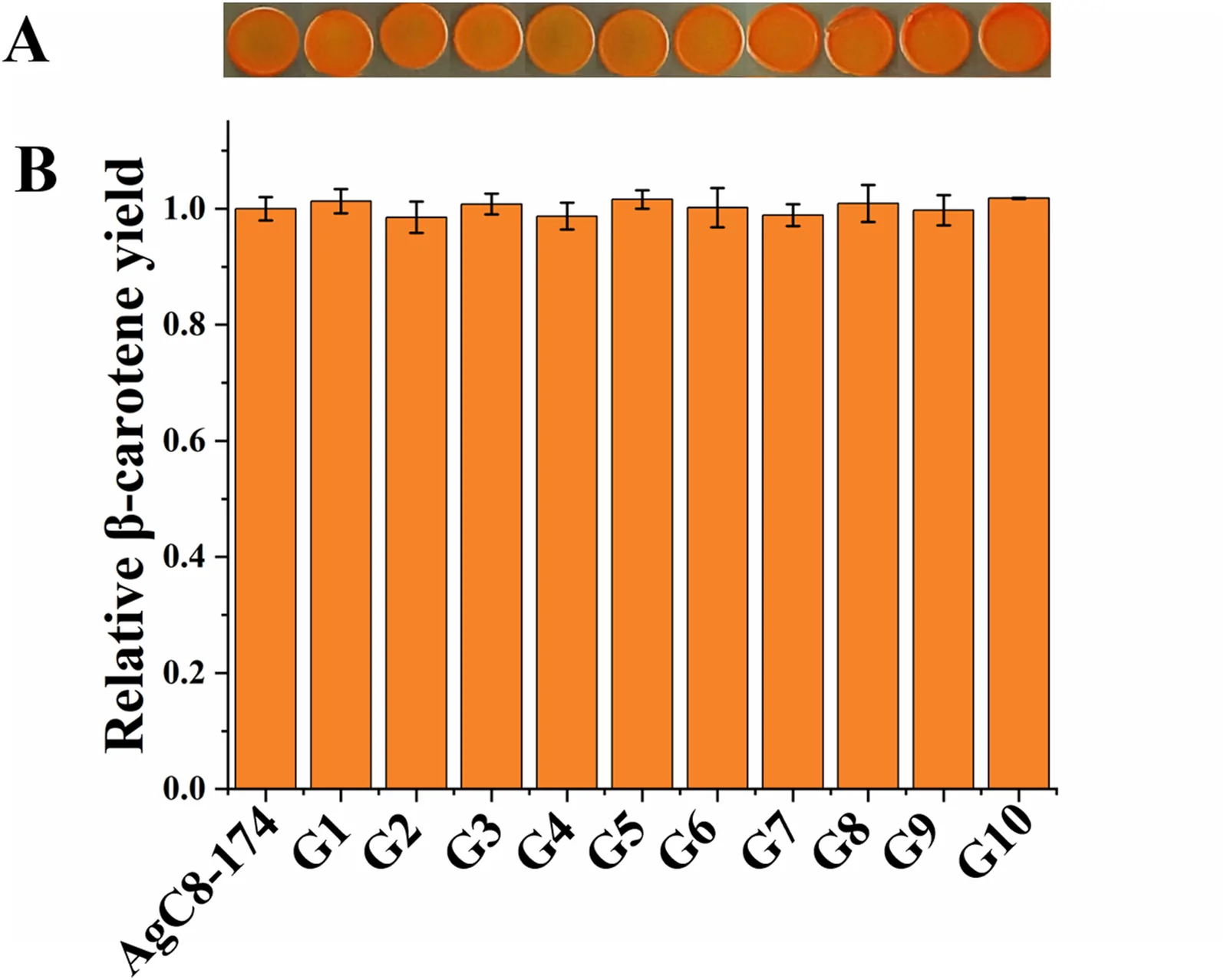
Genetic stability assay of evolved strain AgC8-174. A. Growth assay of the successive generations on YPD agar plates for 72 h. B. Relative β-carotene yield of the successive generations of AgC8-174 (G1 to G10). The β-carotene yield of AgC8-174 was standardized to 1. Error bars represent SD from three independent experiments.

In the future, whole-genome sequencing (WGS) will be performed on the high-yield mutants obtained from successive rounds of AgLAGM evolution. Genomic variations induced by AgLAGM, including key mutation sites, mutation types and preferential genomic regions, will be systematically identified. This analysis aims to screen core genetic variations closely associated with enhanced β-carotene biosynthesis, reveal the genomic mutation characteristics of the AgLAGM strategy, and further elucidate the molecular mechanism underlying the improved strain performance.

## 4 Conclusion

To address the core limitations of traditional CRISPR-mediated genome editing technologies, such as the complex design and synthesis of high-throughput gRNA libraries, poor economy, and limited versatility, this study designed and established a novel genome mutagenesis strategy named AgLAGM, which is based on in vitro transcription of artificially synthesized random gRNA libraries (artificial gRNA_20N_). Using *S*. *cerevisiae* strain C0 as the model strain, after three rounds of iterative mutagenesis, the β-carotene synthesis capacity of strain C0 was increased to 5.8 times that of the original strain, verifying the effectiveness of the AgLAGM system in microbial genetic breeding. For the high-yield β-carotene *S. cerevisiae* mutant strain C7-143, the mutant strain AgC8-174 was obtained through one round of AgLAGM mutagenesis and screening, with a β-carotene yield of 35.78 mg/g CDW, which was 26% higher than that of the original high-yield strain and reached the highest reported level of single-cell β-carotene synthesis in *S. cerevisiae* so far, demonstrating that the system still has high-efficiency evolutionary capacity for high-yield strains.

Compared with traditional CRISPR-mediated genome editing technologies, the AgLAGM strategy completely avoids the drawbacks of plasmid library construction, such as complex procedures, difficult quality control, and copy number limitations, significantly shortens the experimental cycle, and solves the problem of poor compatibility of gRNA libraries among different species, improving the versatility of the technology. This study fully confirms the feasibility and efficiency of the AgLAGM technology, lays a solid theoretical and experimental foundation for its wide application in the field of microbial genetics and breeding, and provides a new technical route for genome-wide random and diversified mutagenesis.

Nevertheless, several limitations of the present system remain to be addressed in future optimization. First, insufficient intracellular stability of gRNA constitutes a major bottleneck. Naked gRNA delivered via electroporation is susceptible to degradation by endogenous yeast ribonucleases, which shortens the functional window for Cas-mediated DNA cleavage [[42], [43], [44]]. This may partially explain why the maximum strain mortality rate reached only 25.3% even at the highest gRNA dosage in this study. Terminal chemical modifications of gRNA, such as 2′-O-methylation and phosphorothioate modification, can be adopted in subsequent studies to enhance nuclease resistance [45]. Second, although the randomness of degenerate bases in the library template is satisfactory, a subset of gRNAs may become non-functional owing to structural defects or impaired Cas-binding affinity [46,47]. Third, while the transient and non-replicable nature of naked gRNA prevents excessive cell death caused by sustained DNA cleavage, RNA electroporation efficiency is markedly affected by the physiological state of cells and electroporation parameters [48]. In addition, the primary screening in this study relied on colony pigmentation. For most metabolites without observable phenotypes, the screening throughput is restricted. Even among high-β-carotene-producing strains, subtle pigment differences between individual isolates require secondary screening via tube-scale fermentation.

## CRediT authorship contribution statement

Xinyi Wang: Investigation, Methodology, Resources, Writing – original draft. Peiyan Ma: Investigation, Methodology, Software. Majda Hanioui: Investigation, Methodology. Zhangnian Wang: Investigation. Qianqian Yu: Data curation, Investigation. Abdul Waheed: Conceptualization, Investigation, Resources. Yan Liu and Zhenglian Xue. Kun Liu: Conceptualization, Investigation, Methodology. Ming Zhao: Data curation, Funding acquisition, Investigation, Supervision, Writing – review & editing.

## Declaration of competing interest

The authors declare that they have no known competing financial interests or personal relationships that could have appeared to influence the work reported in this paper.
