# Supplementary material for: Design and artificial synthesis of gRNA library for genetic breeding of Saccharomyces cerevisiae
Source: Biodes Res. 2026 Sep 28;8(4):100123. doi: 10.1016/j.bidere.2026.100123 (PMC13717876; doi:10.1016/j.bidere.2026.100123)
Supplement: Supplementary file 1 [file mmc1.docx]

**Supplementary Data of**

**Design and artificial synthesis of gRNA library for genetic breeding of *Saccharomyces cerevisiae***

**Xinyi Wang^a,1^, Peiyan Ma^a,1^, Majda Hanioui^a^, Zhangnian Wang^a^, Qianqian Yu^a^, Abdul Waheed^a^, Kun Liu^a,b*^, Yan Liu^a,b^, Zhenglian Xue^a,b^, and Ming Zhao^a,b**^**

^a^Anhui Engineering Laboratory for Industrial Microbiology Molecular Breeding, College of Biology and Food Engineering, Anhui Polytechnic University, 241000 Wuhu, China

^b^Wuhu Green Food Industry Research Institute Co., Ltd, 241000 Wuhu, China

**^*^**Address correspondence to E-mail addresses:

Ming Zhao, zhaom@mail.ahpu.edu.cn; Kun Liu, liuk@ahpu.edu.cn.

^1^These authors contributed equally to this work.

**Table S1. Strains used in this study.**

| Stains | Characteristics | Sources |
| --- | --- | --- |
| *S.cerevisiae* BY4741 | *MATα*, *his3Δ1*, *leu2Δ0*, *met15Δ0*, *ura3Δ0* | This lab |
| Colony1 | HOΔ::P_TEFI_-*crt*l, Gal80Δ::P_HXT7_-*crtYB*;  Gal1-7Δ::P_TEFI_-*tHMG1*, P_HXT7_-*crtE* | This lab |
| C0 | Colonyl, pCas9-NG | This lab |
| C7-143 | CARM strain from the C0 by iterative evolution | This lab |
| C1-2 | AgLAGM strain from the C0 | This study |
| C2-11 | AgLAGM strain from the C1-2 | This study |
| C3-174 | AgLAGM strain from the C2-11 | This study |
| C8-174 | AgLAGM strain from the C7-143 | This study |

**Table S2. Plasmids used in this study.**

| Stains | Characteristics | Sources |
| --- | --- | --- |
| pSCM | gRNA expression plasmid | Addgene |
| pTCL | SpCas9 expression plasmid | Our lab |
| pCas9-NG | SpCas9-NG expression plasmid | Our lab |

**Table S3.** Primers used in this study.

| Primers | From 5’to 3’ |
| --- | --- |
| gRNAL | TAATACGACTCACTATAGGNNNNNNNNNNNNNNNNNNNNGTTTTAGAGCTAGAAATAGCAAGTTAAAATAAGGCTAGTCCGTTATCAACTTGAAAAAGTGGCACCGAGTCGGTGCTTTTTT |
| gRNA-F | TAATACGACTCACTATAGG |
| gRNA-R | AAAAAAGCACCGACTCGGTGCCAC |

**Table S4. The random sequences of gRNA library cassettes.**

**Sequences of 20N (5’-3’)**

GAGGCCTATACGAACAAGTC

GTAATGGTCAGCAGGACCAA

CGCATTGGTCACTCATGCAA

CAGGATATAGCATGAGTATC

CCACGTATAGCAAGTCTATG

GCGTAAGCGATGGATTAAGA

CAAGTCGAGGACTATGACCC

CAAGGGCAACTCAATGGAAA

CTAGCTATCGGGCCGTACCC

GAAAGTAATATATATGACGA

ATGGCACAAATAAAAGTATC

ACTATTCTTGAATCTCGCCA

CGGCAAACGCTAGCCTTAAT

GAAGAACAGACGATAGAGGT

ATGGGGGTTTAGAGCTTGAA

TTAATAAGTAGGTACCTCGT

CTTGTAGAAGAGCAATTAGA

TTTGGTATCTTGACGAGCTC

ACACAACATACGAGCCGGAA

TGGGATGGGTGAGCGACAGT

GGACGAACAGGGTACTTGGG

CCATAGCTCAAGCTAGTCAA

AGAGGGTCACATAAAACTAC

CCGTTAGGGGGCTGACTAAG

CGTAGCGTTCGTAAGGCAGT

GAGGCCCGTTTCAGCTAGAA

TCGAATGTTTCGAGTGTTTC

TAGAACAAAGATATCAATCC

ACACAACATACGAGCCGGAA

CGTGTAAATTGCCCTGAGAA

ACACAACATACGAGCCGGAA

**Figure S1.** Difference in colony color between positive mutants and the starting strain during high-throughput screening. A. The first round iterative evolution of *S. cerevisiae* C0. B. The last round iterative evolution of *S. cerevisiae* C7-143.

**Figure S2.** The second round iterative evolution of *S. cerevisiae* AgC1-151 for β-carotene production based on coloration screening on the plates. All the strains were grown in YPD media at 30 °C for 72 h. Error bars show the standard deviation from three independent experiments.

**Figure S3.** The third round iterative evolution of *S. cerevisiae* AgC2-11 for β-carotene production based on coloration screening on the plates. All the strains were grown in YPD media at 30 °C for 72 h. Error bars show the standard deviation from three independent experiments.

**Figure S4.** The preliminary screening of *S. cerevisiae* C7-143 mutants based on β-carotene production. All the strains were grown in YPD media at 30 °C for 72 h.
